# Human brain structural connectivity matrices–ready for modelling

**DOI:** 10.1038/s41597-022-01596-9

**Published:** 2022-08-09

**Authors:** Antonín Škoch, Barbora Rehák Bučková, Jan Mareš, Jaroslav Tintěra, Pavel Sanda, Lucia Jajcay, Jiří Horáček, Filip Španiel, Jaroslav Hlinka

**Affiliations:** 1 National Institute of Mental Health, Klecany, Czech Republic; 2 Institute for Clinical and Experimental Medicine, Prague, Czech Republic; 3 Institute of Computer Science of the Czech Academy of Sciences, Prague, Czech Republic; 4 Faculty of Electrical Engineering, Czech Technical University in Prague, Prague, Czech Republic

**Keywords:** Neurology, Brain, Network models

## Abstract

The human brain represents a complex computational system, the function and structure of which may be measured using various neuroimaging techniques focusing on separate properties of the brain tissue and activity. We capture the organization of white matter fibers acquired by diffusion-weighted imaging using probabilistic diffusion tractography. By segmenting the results of tractography into larger anatomical units, it is possible to draw inferences about the structural relationships between these parts of the system. This pipeline results in a structural connectivity matrix, which contains an estimate of connection strength among all regions. However, raw data processing is complex, computationally intensive, and requires expert quality control, which may be discouraging for researchers with less experience in the field. We thus provide brain structural connectivity matrices in a form ready for modelling and analysis and thus usable by a wide community of scientists. The presented dataset contains brain structural connectivity matrices together with the underlying raw diffusion and structural data, as well as basic demographic data of 88 healthy subjects.

## Abstract (summary)

**Table:** 

Measurement(s)	Diffusion Weighted Imaging
Technology Type(s)	Magnetic Resonance Imaging
Sample Characteristic - Organism	Homo sapiens
Sample Characteristic - Location	Czech Republic

## Background & Summary

Studying the human brain with magnetic resonance imaging (MRI) has become one of the central avenues in contemporary neuroscience. One of the key questions studied is that of *integration* of information among different brain areas. To estimate the interaction between any two brain areas, MRI provides several different general tools. From the time series of brain activity measured, for instance, using functional magnetic resonance imaging, researchers can estimate the *functional connectivity*, i.e. the (undirected) statistical dependence of the activity of remote regions, or even try to estimate the *effective connectivity*, i.e. the direct effect that one brain region exerts upon another^1^. Yet another, and somewhat more fundamental, piece of the puzzle is provided by the *structural connectivity*, denoting the physical information-carrying connections between the considered neural populations; typically the tracts of white matter containing axons connecting predefined gray matter regions.

Structural connectivity is typically obtained by processing the diffusion-weighted MRI data (DW-MRI, or DWI). The key principle is that the image of each volume element (voxel) is acquired multiple times, each of the images being sensitive to diffusion along a particular spatial axis. From such a set of images, the spatial profile of the preferred directions of diffusion can be estimated. This estimate allows to infer the likely presence and direction of white matter fibers within a given voxel, and to connect the information spatially to generate a simulation of tentative white matter tracts connecting different parts of the brain. The resulting *tractogram* can provide an impressively detailed visualization of the structural connections within the brain, as well as quantitative information on the presence and amount of structural connections between any predefined set of (gray matter) brain regions–the *structural connectivity matrix*.

The structural connectivity matrix represents the network of ‘highways’ the information in the brain can flow along, and as such has been studied extensively^2^. A central question is that of the role that structural connectivity plays in shaping the dynamics of brain activity and, particularly, its relation to the pattern of statistical dependencies between the activity of brain regions - the *functional connectivity matrix*. Following the early works relating structural and functional brain connectivity^3,4^, both variability of results and theoretical modelling and simulations^5,6^ highlighted the role of other factors in the structure-function relationship. The importance and richness of this research area were soon recognized^7^ and motivated a stream of further efforts^8^, and the modelling of static functional connectivity has further extended into the effort to reliably explain three aspects of brain activity dynamics: the spatial properties, temporal dynamics, and spectral features^9^. Modelling functional connectivity of a healthy brain is, however, not the only use of brain structural connectivity data–it has been increasingly used also for modelling of brain disease dynamics, including epilepsy^10,11^, as well as to study deeper characteristics of the structural connectivity itself^12^. This enterprise is thus a promising and growing area of research, calling for the utilization of publicly shared data at a level accessible to data scientists and, generally, researchers across disciplines.

**Fig. 1:**
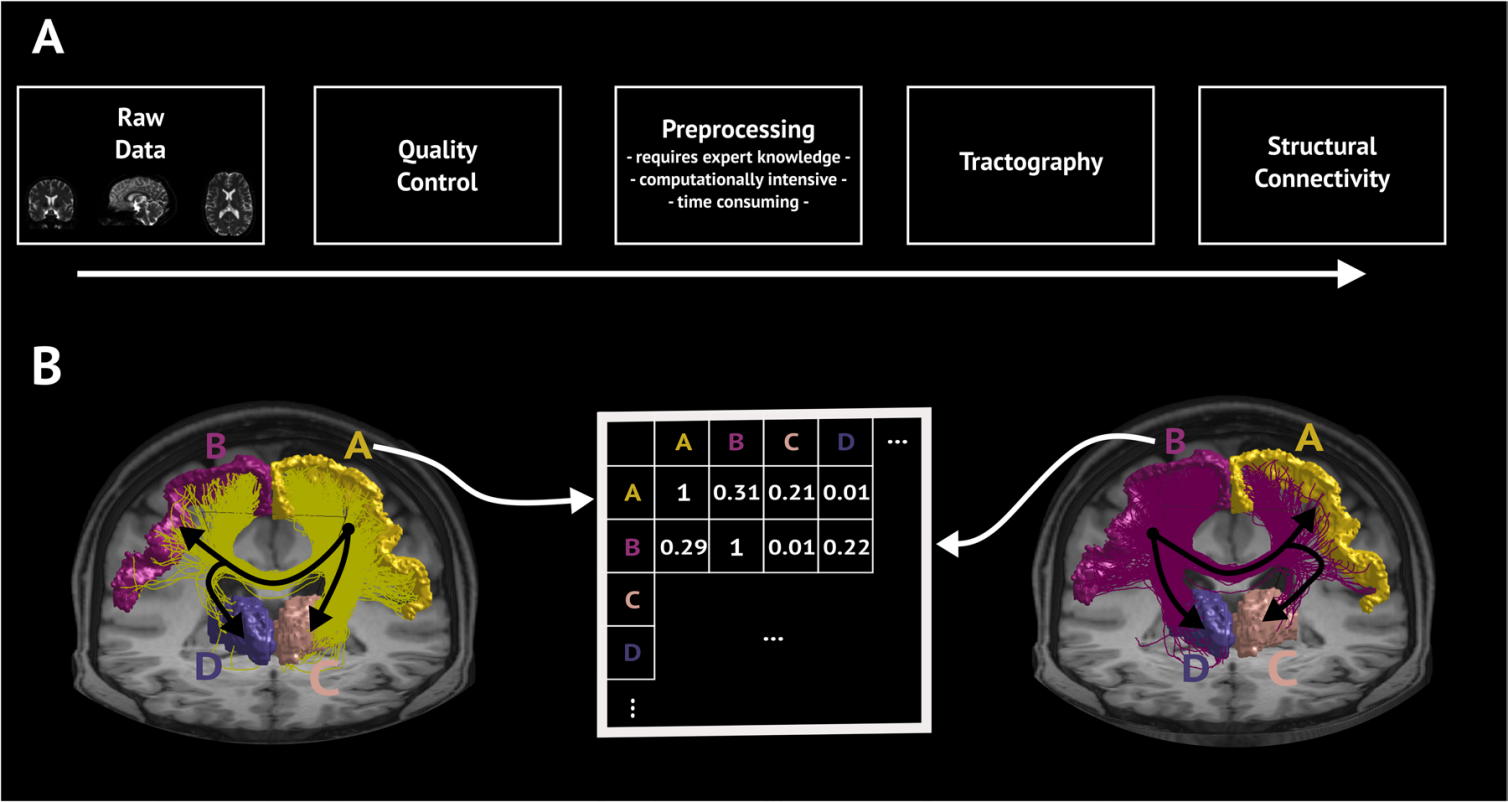
Preprocessing pipeline and the tractography visualization. (**A**) In the upper row, we introduce the key points of structural connectivity construction. (**B**) left: The tracts seeded in region A and leading to three example ROIs B, C, and D are shown. The elements in the first row of the connectivity matrix are proportional to the number of streamlines originating in A and entering the corresponding ROIs. (**B**) right: The same procedure with a focus on the seeds in region B.

However, despite the recent movement for open sharing of neuroimaging data, including large databases such as the Human Connectome Project, there is a limited amount of original large datasets available, in particular in terms of readily available preprocessed connectivity matrices. Indeed, in many cases, only raw data are shared, and the production of structural connectivity matrices from the raw DWI data is a lengthy process requiring expert knowledge and making specific choices. As it is important to provide a plurality of datasets to increase reproducibility and generalizability of the results, we provide here a ready-to-use dataset, consisting of not only raw diffusion and structural data of 88 healthy individuals but also derived brain structural connectivity matrices. The matrices represent the connectivity among 90 cortical regions of interest (ROIs) as defined by the widely used Automatic Anatomical Labeling (AAL) atlas^13^, where every entry of the matrix represents a proportion of tractography streamlines originating in one ROI (given by a row) that enter another ROI (given by a column). The processing is described on a high level in Fig. 1, and in detail in the Methods section.

The connectivity matrices are provided both as a single Matlab readable file and as separate tab-delimited text files. Moreover, the raw diffusion and structural data are also available together with the scripted pipeline that was employed for the generation of structural connectivity matrices. Thus, the users are free to generate structural connectivity matrices in their preferred parcellation scheme. Finally, we also share basic demographic and clinical data of the subjects (gender, age, handedness, education, weight, height, BMI) and the list of AAL ROIs in the order used in the matrices. The data correspond to a control healthy subject cohort from a study on early changes in schizophrenia^14^, and a subset of the connectivity matrices has already been used for previous modelling studies that aimed at modelling chimera states^15^, unihemispheric sleep^16^, or epileptic dynamics^11,17^.

## Methods

### Participants

The data provided here are based on MRI scans of 88 healthy control individuals participating in the Early-Stage Schizophrenia Outcome study^14^ (ESO–a prospective trial conducted in the Prague and Central Bohemia surveillance area, investigating first-episode schizophrenia spectrum subjects). The study was conducted in accordance with the Declaration of Helsinki. The local Ethics Committee of the Prague Psychiatric Center approved the protocol on 29 June 2011 (protocol code 69/11). All participants were informed about the purpose of the study, the experimental procedures, as well as the fact that they could withdraw from the study at any time, and provided written informed consent prior to their participation. The subjects were recruited via local advertisements and excluded if meeting any of the following criteria: personal lifetime history of any psychiatric disorder or substance abuse established by the Mini-International Neuropsychiatric Interview (M.I.N.I.)^18^, any psychotic disorder in first or second-degree relatives, current neurological disorders, a lifetime history of seizures or head injury with altered consciousness, intracranial hemorrhage or neurological sequelae, a history of mental retardation, a history of substance dependence, and any contraindication for MRI scanning.

There are *n* = 88 subjects, 48 female and 40 male, with mean age of 27.7 years (range 18–48 years), mostly right-handed (80 right-handed, 6 left-handed, 2 N/A), with highest completed education: elementary school (*n* = 1), high-school (*n* = 51), bachelor’s degree or higher (*n* = 34), and data not available (*n* = 2). For clinical data of particular subjects, see Supplementary Table 1.

### Strategy of structural connectivity matrices construction

The construction of structural connectivity matrices was based on a connectome generated by probabilistic tractography on diffusion MRI data. We used ROIs from the widely used AAL atlas^13^ (Automated Anatomical Labeling atlas), version ROI_MNI_v4, for the definition of connectivity matrices: the connectivity between two ROIs is based on the number of streamlines in the tractogram beginning in one ROI and terminating in the other ROI. Accurate mapping of the AAL atlas ROIs to the diffusion data space of each subject requires a sufficiently accurate mapping of the diffusion space, where the tractogram is constructed, to the MNI space (Montreal Neurological Institute space), where the AAL ROIs are defined. However, since T1 structural images reflect anatomy in much greater detail than DWI data, they are more suitable for estimation of the mapping to the MNI space than DWI data themselves. Therefore, the estimation of mapping was realized as a two-stage process^19,20^: affine mapping of structural T1 images to MNI space and a rigid-body mapping between the T1 structural data and the DWI data, both for each subject. Further, as tractography is designed to follow white matter tracts and does not result in meaningful streamlines in gray matter, white matter masks must be estimated. Here, we utilize the fact that, despite AAL being a gray-matter atlas, its ROIs also reach into white matter, and voxels where this happens can be used for tractography. A diagram of the entire data processing is shown in Fig. 4.

### MRI data acquisition

#### Scanner

We performed the MRI scanning at the Institute for Clinical and Experimental Medicine in Prague, on a 3 T Trio Siemens scanner (Erlangen, Germany). A 12-channel head coil was used, software version syngo MR B17.

#### DWI data acquisition

DWI data were acquired by a Spin-Echo EPI sequence with TR/TE = 8300/84 ms, matrix 112 × 128, voxel size 2 × 2 × 2 mm^3^, b-value 0 and 900 s/mm^2^ in 30 diffusion gradient directions, 2 averages, bandwidth 1502 Hz/pixel, GRAPPA acceleration factor 2 in phase-encoding direction, reference lines 24, prescan normalize off, elliptical filter off, raw filter on – intensity: weak, acquisition time 9:01.

#### T1 acquisition

T1 3D structural image was acquired by using the magnetization prepared rapid acquisition gradient echo (MPRAGE) sequence with (TI – inversion time) TI/TR/TE = 900/2300/4.63 ms, flip angle 10°, 1 average, matrix 256 × 256 × 224, voxel size 1 × 1 × 1 mm^3^, bandwidth 130 Hz/pixel, GRAPPA acceleration factor 2 in phase-encoding direction, reference lines 32, prescan normalize on, elliptical filter on, raw filter off, acquisition time 5:30.

### DWI preprocessing and processing

- DWI data were visually inspected to check their quality. Subjects with excessive image artifacts were excluded.
- Individual DWI volumes of each subject were inspected. Volumes containing artifacts (k-space spikes, signal void due to movement, etc.) were excluded from further processing.
- The DWI data were preprocessed using FSL tools^21^ version 5.0.7. Movement and eddy-current distortions were corrected by affine registration using FLIRT. A dedicated dti_preprocess^22^ script, version 1.8, was used for this purpose.
- DWI images were skull-stripped using FSL BET^23^.
- Diffusion parameters were obtained by Bayesian estimation using the BEDPOSTX tool^24^.

### Subject DWI space to MNI template space registration

#### Terminological note

Within the community using SPM software, the term “spatial normalization”, or even more imprecise “normalization”, is commonly used to refer to the process of estimation of a mapping and/or applying this mapping. Within communities using other neuroimaging software, the term “registration” is more commonly used.

- T1 images were skull-stripped using FSL BET.
- Skull-stripped structural T1 images were registered to the MNI space for each subject using the FSL FLIRT tool. The FSL T1 MNI template with 2 mm resolution was used for the affine registration with 12 DOFs (degrees of freedom).
- Skull-stripped T1 and DWI images of each subject were registered using rigid body transformation (6 DOFs). This is sufficient as we register two images of the same subject. The registration was performed by the FSL epi_reg script, which uses BBR (Boundary-Based Registration) cost function, which was shown to be more accurate than single-stage methods^25^.
- The transformation matrices from the two steps above were combined to transform AAL ROI masks from the standard MNI space to the DWI space of each subject.
- The transformed AAL masks were further restricted by white matter masks. Those were obtained by FSL FAST^26^ segmentation of the skull-stripped T1 images of the respective subjects, and further restricted by brain masks derived from the subjects’ DWI data. This is because the skull stripping of T1 images had to be conservative to prevent the exclusion of genuine parts of the brain, and therefore also contained some non-brain structures, which can be removed by the DWI derived brain mask.

### Probabilistic tractography and connectivity matrices

- Probabilistic tractography using voxel-wise diffusion parameters estimated by PROBTRACKX was performed. The white matter masks were used for the spatial restriction of streamlines. In particular, for each AAL atlas ROI, the FSL command probtrackx2 with default parameters was used to generate 5000 streamlines spreading from each voxel to the rest of the brain.
- The connectivity between two ROIs is estimated from the number of streamlines seeded in one ROI (in any of its voxels) that enter the other ROI. (A streamline is counted in all ROIs it visits.) The AAL ROIs located in the cerebellum were not used, so the matrices have 90 × 90 elements.
- The connectivity matrices were normalized by the number of voxels in the seed ROI and the number of streamlines per voxel (5000). This resulted in a “connectivity probability” matrix, where each element is an estimate of the probability of reaching the target ROI via a randomly chosen streamline from the seed ROI (Fig. 1).

## Data Records

The structural connectivity matrices, together with all the source data necessary for their replication and the processing pipeline in the form of ordered scripts, are publicly available on the Open Science Framework (OSF): (10.17605/OSF.IO/YW5VF)^27^, under a Creative Commons Attribution 4.0 International License. Detailed information about the scripts is provided in the Code Availability section.

### Source data

The data/ folder contains the source data from which the structural connectivity matrices were derived. All MRI data are stored as compressed (gzipped) NIfTI-formatted images. To preserve our subjects’ anonymity, raw structural data are not shared. The data of each subject are contained in an individual subfolder named according to their anonymized numerical identifier (e.g. S001), and further divided into two subfolders reflecting the two main categories of source data.

The subfolder with diffusion data (data/S001/dti_tra_epi_2D_MDDW30/) contains three to four files, namely the raw diffusion data (S001_diff.nii.gz), b-vectors (S001_diff.bvec), b-values (S001_diff.bval), and, where applicable, a text file with the numbers of volumes to be excluded (S001_volumesToExclude.txt).

The subfolder with structural data (data/S001/t1_sag_mpr/) contains data derived from the raw structural image and necessary for further steps of the processing, namely the skull-stripped T1 (S001_struct_ori_crop_brain.nii.gz), the subject T1-DWI transformation matrix (S001_data2struct.mat), the white matter mask in subject T1 space (S001_data2struct_fast_wmseg.nii.gz).

### Structural connectivity matrices

**Fig. 2:**
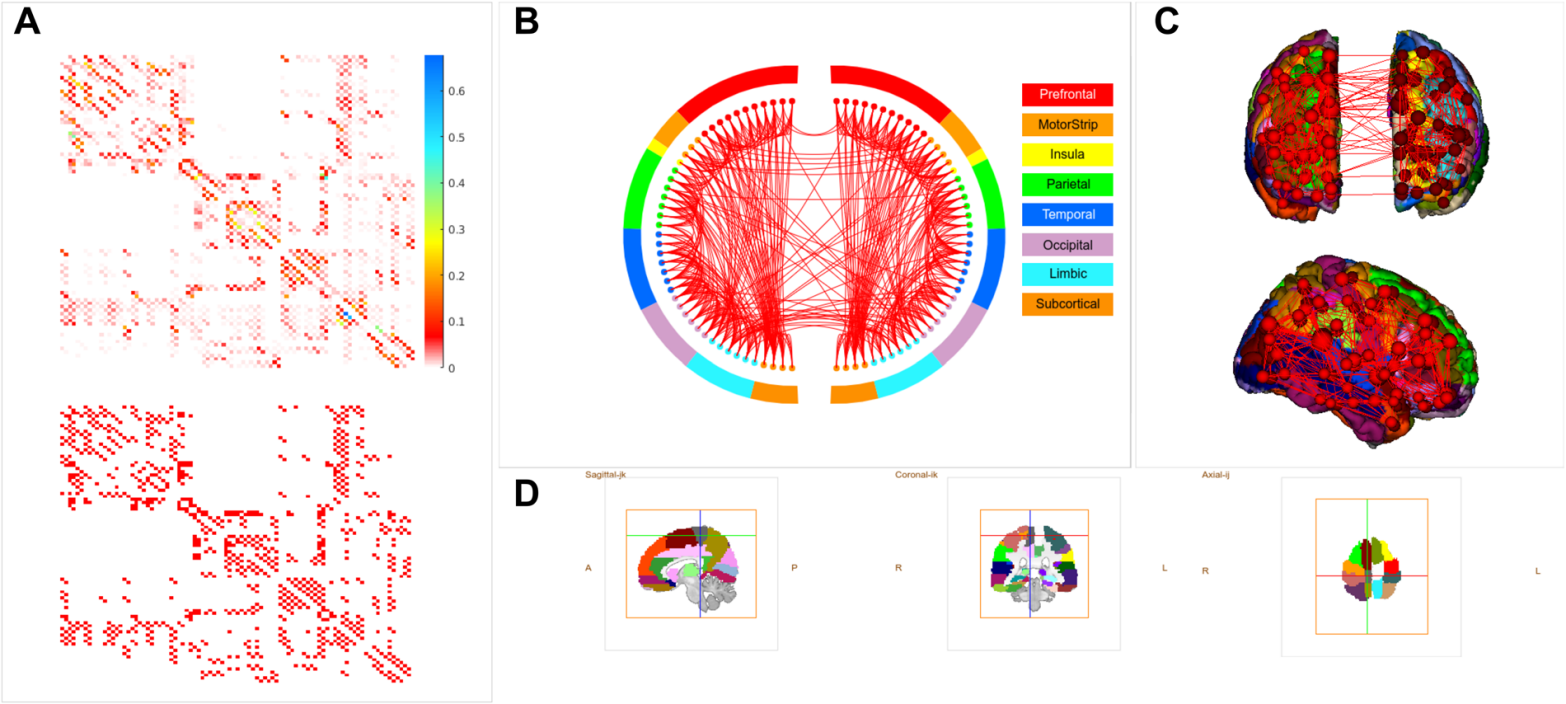
Average structural connectivity. (**A**) Adjacency matrix of the average structural connectivity (up) and the same matrix thresholded at 0.01 (see the following subfigures). (**B**) Network representation of structural connectivity. (**C**) Connectivity mapped on the brain surface. (**D**) Used brain parcellation in sagittal, coronal, and axial view.

The SC matrices (for visualization, see Fig. 2) are shared in two formats. The structural_connectivity_matrices/ folder contains one.csv file with the individual SC matrix of each subject (e.g. S001.csv). The single Matlab table file (SCmatrices88healthy.mat) contains all of the individual SC matrices in the order in which subjects are listed in clinics.csv (see also Supplementary Table 1.

For every subject there is a 90 × 90 matrix of float numbers representing the “connectivity probability” from one ROI (given by the row index) to another ROI (given by the column index) from the AAL atlas. For convenience, the 90 cortical ROIs used are listed in AAL_regions.csv, in the order used in the matrices. In particular, a given matrix entry is a proportion of streamlines seeded in the first ROI which reached the other ROI. All diagonal entries are zeros. Since the streamlines can either miss all the other ROIs entirely or pass multiple ROIs, the off-diagonal values in rows/columns may sum to a number both lower than one or greater than one. While the matrices are close to symmetric as a result of the non-directional representation of the white matter tracts obtained from diffusion MRI, they are not entirely symmetrical and no symmetrization was applied on them. For more discussion, see the Technical Validation section.

Finally, the folder structural_connectivity_matrices_not_normalized contains SC matrices for all subjects before the normalization, that is, the raw number of streamlines connecting two ROIs.

## Technical Validation

**Fig. 3:**
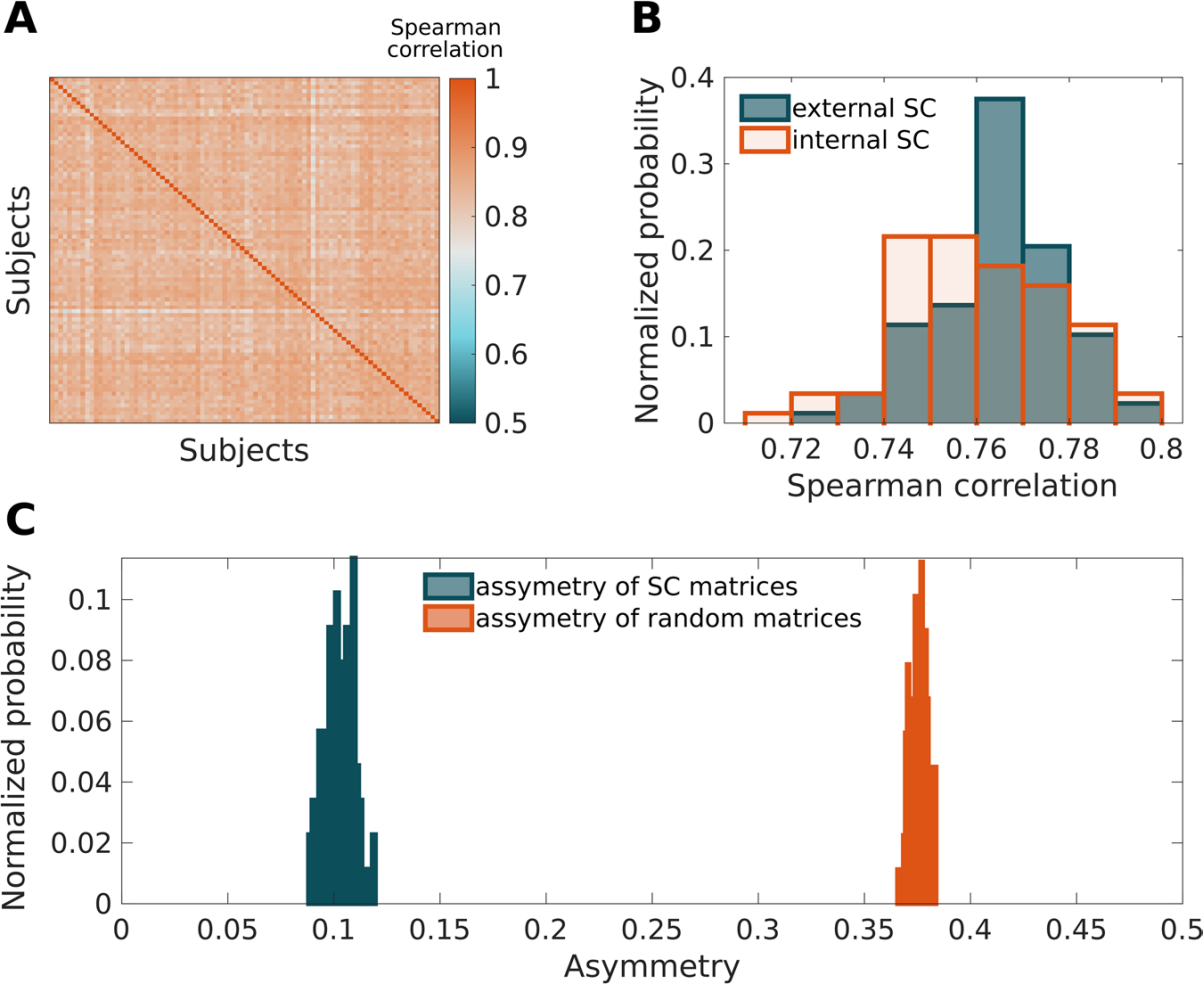
The results of validation. (**A**) Correlation coefficients between individual structural connectivity matrices for all pairs of subjects in the dataset; (**B**) histogram of correlation of all subjects with the external SC matrix; (**C**) Histogram of the asymmetry of the provided SC matrices (blue) and of the asymmetry of the same number of random matrices with the same value distribution.

Quality control of diffusion data has not yet been standardized and largely relies on visual inspection following individual steps of the preprocessing pipeline^28^. As mentioned above, we ensured that the volumes containing excessive image artifacts were discarded and, overall, we checked all steps of the processing and took precautions to prevent any gross or systematic errors. Here, we present two analyses to increase confidence that the data were processed correctly. To further screen for the possibility that some technical failure of the processing occurred in some of the subjects, we correlated SC matrices of all subjects and produced a similarity matrix of the SC matrices (Fig. 3A) which, in all cases, achieved a significant degree of correlation.

**Fig. 4:**
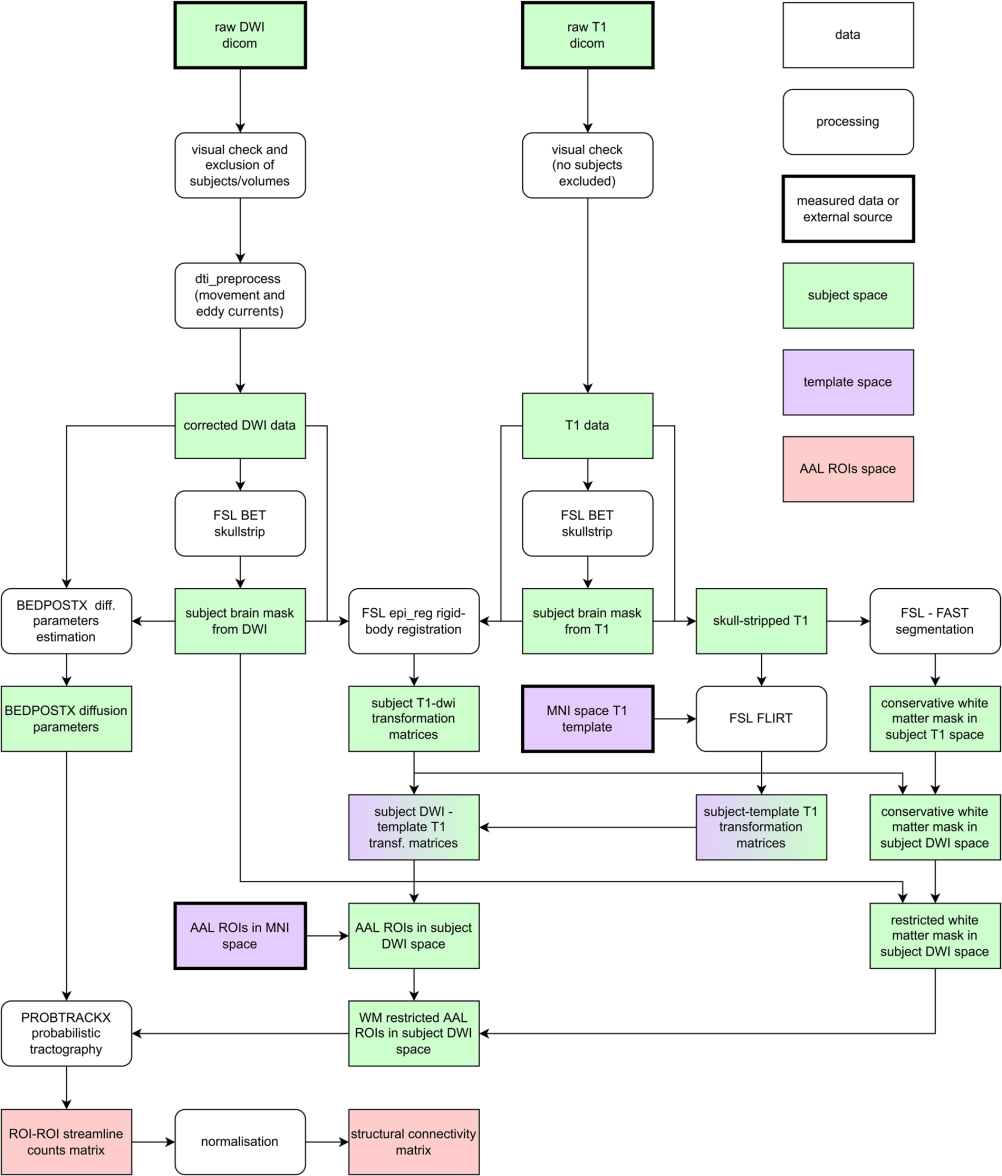
A detailed diagram of the whole data processing. Rectangles with sharp corners represent data, and those with rounded corners represent processing steps. Colors indicate if the data are represented in the individual subjects’ space (green), in the MNI template space (violet), or in the space of AAL atlas ROI indices (red).

The mutual correlations provide evidence of internal consistency in our data; we further provide a comparison to an external reference. In particular, we compared our data with a structural connectivity matrix constructed from publicly available tractography^29^. In this case, the acquisition device, protocol, and tractography construction methods differed. However, despite these technical differences, our data achieved, on average, a correlation of 0.76 to this external dataset (Fig. 3B).

In this last part of the technical validation, we assessed the measure of asymmetry of the presented SC matrices. As our construction of SC matrices does not enforce their symmetry in any direct artificial way, the matrices are not perfectly symmetrical. In modelling applications, it is common to symmetrize SC matrices simply by calculating *A*_*sym*_ = (*A* + *A*′)/2 (see e.g.^30^), where the prime denotes matrix transposition. It can be argued that the asymmetry contains some possibly usable information, but it must be stressed that it does not carry any information about the directionality of the white matter fibers, as the matrices originate from principally non-directed diffusion tensor data. An example of a situation in which the asymmetry carries potentially relevant information is when e.g. an ROI *x* is large and connected to multiple other ROIs, while another ROI *y* is small and connected almost exclusively to *x*. In that case, the element *A*_*x,y*_ will be small (streamlines distributed into multiple other ROIs) and the element *A*_*y,x*_ will be close to 1 (most of the streamlines going to *x*).

To quantify the degree of asymmetry, for every matrix *A* we computed *asym*(*A*) = ||*A*−*A*′||/||*A* + *A*′||; where ||*X*|| denotes the Frobenius norm of a matrix *X*. In this case, the symmetric matrix would have *asym(A)* equal to zero. The average of asymmetry measures in the dataset was 0.103 (std: 0.007) and the distribution across the data is depicted in Fig. 3. While the matrices are not perfectly symmetric, the measure of asymmetry in the data is significantly smaller than in the same number of random matrices with the same value distribution (p < 0.001).

## Usage Notes

As mentioned in the introduction, there is a wide range of possible research uses for structural connectivity data. While, for some of these, specialized software may be useful, we made sure that there is a low entry threshold in that the structural connectivity matrices are ready for direct usage without any further requirements. The data are thus already fully preprocessed and saved in the form of tab-delineated text files loadable by using arbitrary software or scripting/programming language. For the convenience of the substantial part of the neuroimaging community using Matlab, the data can also easily be loaded using Matlab, as one file.

Specific examples of uses of the data include the analysis of the graph-theoretical properties of the brain structural network; to this end, among other tools, the Brain Connectivity Toolbox^31^ or BioImage Suite^32^ (used to create Fig. 2) might serve as a useful entry tool. Another important example is using the structural connectivity to model dynamics of brain activity and functional connectivity, either by mass models primarily focusing on reproducing BOLD signal features^33^ or by more detailed large-scale models^34^. Due to the simplicity of the format and the relatively straightforward interpretation of the presented matrices creating the underlying connectome either for the plethora of existing toolkits (e.g.^35–39^) or custom-written code should not pose a challenge for modellers. Apart from conducting novel analyses, we encourage researchers to try to replicate and extend any of the results that have been reported by the use of (a subset of) the current dataset^11,15,16^, or other results in the literature that used structural connectivity data derived with the same^30^ or other methodologies to test the robustness of the previously reported analyses and simulation results.

For analysis of structural brain connectivity matrices, as well as its use for modelling brain activity, it is important to keep in mind some inherent methodological limitations and challenges of the process of estimation of the brain connectivity structure using diffusion-weighted imaging. We refer the reader to a detailed review of these^40^, however, point out here at least those most relevant for interpreting the results of such enterprise. The first point is that, albeit the structural connectivity obtained by the standard methods is directed (i.e. not symmetrical), the interpretation of the direction of these links is not straightforward and the matrices are thus most commonly symmetrized before being used. We comment more on this point in the Technical Validation section. Secondly, due to the inherent difficulty of the tracking of the path of white matter tracts through bundles where they intertwine with other tracts, the interhemispheric connectivity between contralateral hemispheres is typically underestimated in SC matrices. Conversely, tractography-based methods of estimating structural connectivity tend to suffer from false positives, and some thresholding of the matrices might thus be warranted^41^.

## Supplementary information

Supplementary Table 1

## Data Availability

All code used for the generation of the structural connectivity matrices from the raw diffusion and structural data is also available at 10.17605/OSF.IO/YW5VF^27^, under scripts/. All scripts use FSL tools, version 5.0.7.0^21^. The four custom scripts are numbered in order of execution. • 1_dti_preprocess excludes DWI volumes with artifacts using excludeVols, performs preprocessing of DWI data using preprocess_dti_Takuya_2013 (also available at http://www.bic.mni.mcgill.ca/thayashi/dti.html^22^ as dti_preprocess), and submits preprocessed data to the BEDPOSTX tool (estimation of diffusion parameters). Note that the optional averaging of data using dti_avg is not compatible with exclusion of volumes with artifacts, and was not included in our processing pipeline. • 2_dti_reg performs skull-stripping of raw T1 images, the registration of DWI and T1 images, and the registration to MNI space. • 3_dti_track2 prepares AAL ROI masks in subject DWI space and performs probabilistic tractography. It requires the AAL template (ROI_MNI_V4.nii) and a text file with the mask IDs of the 90 cortical ROIs to be represented in the structural connectivity matrix (ROI_MNI_V4_90.txt), provided under scripts/AAL/. • 4_dti_get_conn2 creates the structural connectivity matrix.

## References

[1] Friston K (1994). Functional and effective connectivity in neuroimaging: A synthesis. Human Brain Mapping.

[2] Griffa A, Baumann PS, Thiran JP, Hagmann P (2013). Structural connectomics in brain diseases. NeuroImage.

[3] Ghosh A, Rho Y, McIntosh AR, Kötter R, Jirsa VK (2008). Noise during rest enables the exploration of the brain’s dynamic repertoire. PLoS Computational Biology.

[4] Honey CJ (2009). Predicting human resting-state functional connectivity from structural connectivity. Proceedings of the National Academy of Sciences of the United States of America.

[5] Daffertshofer, A. & van Wijk, B. C. On the influence of amplitude on the connectivity between phases. *Frontiers in Neuroinformatics* **5**, 10.3389/fninf.2011.00006 (2011). 10.3389/fninf.2011.00006 PMC3139941 21811452

[6] Hlinka J, Coombes S (2012). Using computational models to relate structural and functional brain connectivity. European Journal of Neuroscience.

[7] Park H-J, Friston K (2013). Structural and functional brain networks: From connections to cognition. Science.

[8] Straathof M, Sinke MRT, Dijkhuizen RM, Otte WM (2019). A systematic review on the quantitative relationship between structural and functional network connectivity strength in mammalian brains. Journal of Cerebral Blood Flow & Metabolism.

[9] Cabral J, Kringelbach ML, Deco G (2017). Functional connectivity dynamically evolves on multiple time-scales over a static structural connectome: Models and mechanisms. NeuroImage.

[10] Jirsa VK (2017). The Virtual Epileptic Patient: Individualized whole-brain models of epilepsy spread. NeuroImage.

[11] Gerster M (2021). Patient-specific network connectivity combined with a next generation neural mass model to test clinical hypothesis of seizure propagation. Frontiers in Systems Neuroscience.

[12] Zhang, F. *et al*. Quantitative mapping of the brain’s structural connectivity using diffusion MRI tractography: A review. *Neuroimage*, 118870, 10.1016/j.neuroimage.2021.118870 (2022). 10.1016/j.neuroimage.2021.118870 PMC9257891 34979249

[13] Tzourio-Mazoyer N (2002). Automated anatomical labeling of activations in SPM using a macroscopic anatomical parcellation of the MNI MRI single-subject brain. NeuroImage.

[14] Melicher T (2015). White matter changes in first episode psychosis and their relation to the size of sample studied: A DTI study. Schizophrenia research.

[15] Chouzouris T (2018). Chimera states in brain networks: Empirical neural vs. modular fractal connectivity. Chaos.

[16] Ramlow, L. *et al*. Partial synchronization in empirical brain networks as a model for unihemispheric sleep. *EPL* **126**, 10.1209/0295-5075/126/50007 (2019).

[17] Gerster M (2020). FitzHugh-Nagumo oscillators on complex networks mimic epileptic-seizure-related synchronization phenomena. Chaos.

[18] Lecrubier Y (1997). The Mini International Neuropsychiatric Interview (MINI). A short diagnostic structured interview: reliability and validity according to the CIDI. European Psychiatry.

[19] Jenkinson M, Smith S (2001). A global optimisation method for robust affine registration of brain images. Medical Image Analysis.

[20] Jenkinson M, Bannister P, Brady J, Smith S (2002). Improved optimisation for the robust and accurate linear registration and motion correction of brain images. NeuroImage.

[21] Jenkinson M, Beckmann C, Behrens T, Woolrich M, Smith S (2012). FSL. NeuroImage.

[22] Hayashi, T. DTI preprocess script. *web*, http://www.bic.mni.mcgill.ca/thayashi/dti.html (2013).

[23] Smith SM (2002). Fast robust automated brain extraction. Human Brain Mapping.

[24] Behrens TEJ, Berg HJ, Jbabdi S, Rushworth MFS, Woolrich MW (2007). Probabilistic diffusion tractography with multiple fibre orientations: What can we gain. NeuroImage.

[25] Greve DN, Fischl B (2009). Accurate and robust brain image alignment using boundary-based registration. NeuroImage.

[26] Zhang Y, Brady M, Smith S (2001). Segmentation of brain MR images through a hidden Markov random field model and the expectation-maximization algorithm. IEEE Trans Med Imag.

[27] Skoch A (2021). Open Science Framework.

[28] Soares J, Marques P, Alves V, Sousa N (2013). A hitchhiker’s guide to diffusion tensor imaging. Frontiers in Neuroscience.

[29] Tahedl MBATMAN (2020). Open Science Framework.

[30] Cabral J (2013). Structural connectivity in schizophrenia and its impact on the dynamics of spontaneous functional networks. Chaos.

[31] Rubinov M, Sporns O (2010). Complex network measures of brain connectivity: Uses and interpretations. NeuroImage.

[32] Papademetris, X. *et al*. BioImage Suite: An integrated medical image analysis suite: An update. *The Insight Journal* (2006). PMC4213804 25364771

[33] Aquino KM (2022). On the intersection between data quality and dynamical modelling of large-scale fMRI signals. Neuroimage.

[34] Shimoura, R. O. *et al*. Building a model of the brain: From detailed connectivity maps to network organization. *The European Physical Journal Special Topics* 1–23, 10.1140/epjs/s11734-021-00152-7 (2021).

[35] Tikidji-Hamburyan RA, Narayana V, Bozkus Z, El-Ghazawi TA (2017). Software for brain network simulations: A comparative study. Frontiers in Neuroinformatics.

[36] Sanzleon P (2013). The Virtual Brain: A simulator of primate brain network dynamics. Frontiers in Neuroinformatics.

[37] Heitmann S, Aburn MJ, Breakspear M (2018). The Brain Dynamics Toolbox for Matlab. Neurocomputing.

[38] Sherfey JS (2018). DynaSim: A MATLAB toolbox for neural modeling and simulation. Frontiers in Neuroinformatics.

[39] Dai K (2020). Brain Modeling ToolKit: An open source software suite for multiscale modeling of brain circuits. PLOS Computational Biology.

[40] Schilling KG (2019). Challenges in diffusion MRI tractography–Lessons learned from international benchmark competitions. Magnetic Resonance Imaging.

[41] Maier-Hein KH (2017). The challenge of mapping the human connectome based on diffusion tractography. Nature Communications.

